# *Hes5.9* Coordinate FGF and Notch Signaling to Modulate Gastrulation via Regulating Cell Fate Specification and Cell Migration in *Xenopus tropicalis*

**DOI:** 10.3390/genes11111363

**Published:** 2020-11-18

**Authors:** Xiao Huang, Liyue Zhang, Shanshan Yang, Yongpu Zhang, Mingjiang Wu, Peichao Chen

**Affiliations:** 1 College of Life Sciences, Zhejiang University, Hangzhou 310058, China; 2 The First affiliated Hospital, College of Medicine, Zhejiang University, Hangzhou 310003, China; zhangliyue@zju.edu.cn; 3 College of Life and Environmental Science, Wenzhou University, Wenzhou 325035, China; syang0966@gmail.com (S.Y.); zhangyp@wzu.edu.cn (Y.Z.); wumingjiangwz@163.com (M.W.)

**Keywords:** *Hes5.9*, FGF, Notch, gastrulation, cell fate specification, cell migration

## Abstract

Gastrulation drives the establishment of three germ layers and embryonic axes during frog embryonic development. Mesodermal cell fate specification and morphogenetic movements are vital factors coordinating gastrulation, which are regulated by numerous signaling pathways, such as the Wnt (Wingless/Integrated), Notch, and FGF (Fibroblast growth factor) pathways. However, the coordination of the Notch and FGF signaling pathways during gastrulation remains unclear. We identified a novel helix–loop–helix DNA binding domain gene (*Hes5.9*), which was regulated by the FGF and Notch signaling pathways during gastrulation. Furthermore, gain- and loss-of-function of *Hes5.9* led to defective cell migration and disturbed the expression patterns of mesodermal and endodermal marker genes, thus interfering with gastrulation. Collectively, these results suggest that *Hes5.9* plays a crucial role in cell fate decisions and cell migration during gastrulation, which is modulated by the FGF and Notch signaling pathways.

## 1. Introduction

Embryogenesis is an extremely complex process. That fertilized eggs are developed to shaped individuals, rather than mounds of pluripotent cells, is largely due to a short period termed gastrulation, which comprises a great many critical events, such as morphogenetic movements, specification of body axes and germ layer, and body plan establishment. By temporal-spatial coordination of cell specification and dynamic cell movement, a three germ layer body plan is established, accompanied by embryonic axis formation, during gastrulation. Primordial organs or tissue rudiments are then formed along the axis and ultimately develop into functional organs or tissues. Thus, gastrulation plays pivotal roles during embryogenesis and organogenesis. During gastrulation, the most dramatic morphogenetic change takes place on mesodermal cells, which are specified and spread between ectoderm and endoderm. To date, research from represented model organisms has revealed that numerous signaling pathways, such as the Wnt (Wingless/Integrated), FGF (Fibroblast growth factor), BMP (Bone morphogenetic protein), Notch, and TGF-β (Transforming growth factor-beta)/Nodal signaling pathways [1,2], are involved in mesoderm specification and movement. However, the molecular mechanisms and temporal-spatial orchestrations of these signaling still remain largely obscure.

Although the manifestations of cell movement during gastrulation differ between phyla, some evolutionarily conserved movements, such as epiboly, internalization, convergence, and extension can be characterized [3]. The amphibian model *Xenopus* species contributes greatly to understanding the molecular and cellular mechanisms during gastrulation. In *Xenopus*, gastrulation is typically driven by convergent extension, intercalation, and cell migration. Convergent extension plays a pivotal role in elongating the dorsal marginal zone along the anteroposterior axis [4], which drives the axial and paraxial mesodermal tissues, narrowing (convergence) and lengthening (extension), and also results in blastopore closure and anteroposterior body axis elongation [5,6]. The FGF signaling is reported to modulate multiple developmental processes during early embryogenesis [7,8,9]. During *Xenopus* gastrulation, FGF signaling plays an integral role in the induction and maintenance of mesoderm [10], and also regulates morphogenetic movements directly or indirectly [7]. However, it remains equivocal how FGF signaling interplays with other signaling pathways during the induction, maintenance, and specification of different mesodermal regions.

The Notch signaling pathway is conserved in metazoans, which has usually been shown to modulate cell fate decision and form the boundary between embryonic tissues [11]. The Notch receptors bind to their adjacent cells’ ligands (Deltas), resulting in cleavage and release of the intracellular domain of Notch (NICD), which then are translocated into the nucleus and interact with CSL (a DNA-binding protein named CBF1 in humans; Su(H) in *Drosophila*; LAG1 in *C. elegans*) to form a trans-activating complex on the promoters of downstream target genes. The hairy-enhancer of split (Hes)/hairy-enhancer of split related with YRPW motif (Hey) family members [12] are not all direct effector genes of the Notch signaling pathway. They encode basic helix–loop–helix (bHLH) transcriptional repressors that control cell fate decisions and cell population expansion. For example, mouse Hes1 and Hes5 can inhibit neuronal differentiation while promoting the proliferation of neural progenitors in the embryonic brain [13]. In *Xenopus*, Hes3 [14] and Hes4 (Hairy2) [15] mediate the Notch signaling on neural crest induction on the ridge of the neural plate board. Interestingly, there is clear evidence that Notch signaling is active from the beginning of gastrulation in *Xenopus*. For example, components of the Notch pathway such as Notch, and Serrate-1 Su(H)1 are present maternally and zygotically [16,17], ligands such as Delta-1 and Delta-2 are present in the marginal zone as a ring encircling the blastopore at early gastrula [18,19,20], and more importantly, Notch signaling has been reported to be involved in the segregation and boundary formation of the three germ layers in *Xenopus* during gastrulation [21].

Intriguingly, on integration with other signaling pathways, gastrulation is cellular context responsive, and delicately fine-tuned by Notch signaling [22,23]. The existence has been reported of coordination between FGF and Notch signaling in the establishment of the proper periodicity of vertebrate somite [22,24], ear development [25], and sensory neuron formation [26], via specific target genes. Thus, whether FGF and Notch signaling coordinate during gastrulation, and what are the key targets, are of great interests for elucidating the molecular events and the underlying mechanism of specification and patterning of the germ layers.

We previously screened the differential expression transcripts under the influence of FGF signaling in frog *Xenopus tropicalis* gastrula by treating with FGFR (Fibroblast growth factor receptor) inhibitor SU5402. A novel basic helix-loop-helix gene (*Hes5.9*) has been isolated. In this work, we report the expression and mainly characterize the function of *Hes5.9* by microinjection of synthetic *Hes5.9* mRNA, and antisense oligonucleotides respectively. In general, our results demonstrate that *Hes5.9* may function as a transcriptional factor and be regulated by the FGF and Notch signaling, which is critical for cell fate determination and gastrulation during early embryonic development.

## 2. Materials and Methods

### 2.1. Animal Ethics and Embryo Manipulation

*Xenopus tropicalis* (Nigerian) were purchased from NASCO (USA), then bred and maintained in our lab. All animal procedures were performed in full accordance with the requirements of the Regulation on the Use of Experimental Animals in Zhejiang Province. This work was specifically approved by the Animal Ethics Committee of the School of Medicine, Zhejiang University (ETHICS CODE Permit NO. 14887, issued by the Animal Ethics Committee in the School of Medicine, Zhejiang University). In brief, ovulation was induced by injection of human chorionic gonadotropin (HCG) into the dorsal lymph sac of mature frogs; male and female frogs were injected with 150 and 200 units of HCG, respectively. The embryos were dejellied by 2% cysteine (pH 8.0) and then cultured in 0.1× Marc’s modified ringer solution (MMR). Developmental stages were assessed according to Nieuwkoop and Faber (1994). For drug treatment, we incubated the embryos in 0.1× MMR solution containing 20 μM SU5402 (Santa Cruz) or Dimethyl sulfoxide (DMSO, Sigma) at stage 8, and then removed the solution at stage 11.

### 2.2. Multiple Sequence Alignment and Phylogenetic Tree Construction

The relative protein sequences were retrieved from NCBI, Xenbase, and Ensemble databases, and then aligned via DNAMAN (Lynnon Biosoft, CA, USA) with default parameters. Phylogenetic trees were constructed by neighbor-joining algorithm, and displayed via DNAMAN. The proximal elements of promoters were obtained as described [27,28], and predicted at http://jaspar.genereg.net.

### 2.3. RNA Extraction, Reverse Transcription-PCR, and cDNA Cloning

Different developmental stage embryos were collected. After homogenizing with RNAiso plus (Takara), chloroform was added. The homogenate was then centrifuged and divided into three layers, the total RNA was precipitated from the upper aqueous layer with isopropanol, and impurities removed with 70% ethanol. After that, the RNA degradation and contamination were detected by 1% agarose gel electrophoresis; 1 μg of total RNA was reversed to cDNA by using oligonucleotide (dT)-tailed primer and Reverse Transcriptase M-MLV (Takara), 10 μL reaction volume including l μg total RNA, 50 μM Oligo(dT)12–18 primer, 5× M-MLV Buffer, 10 mM dNTP Mixture, 40 U/μL RNase Inhibitor, 200 U/μL RTase M-MLV, and RNase-free water, the mixture was incubated at 42 °C for 1 h, and then the reaction was stopped by heating at 70 °C at 15 min. The primers with restriction sites for SmaI and NotI were designed to amplify the full length of *Hes5.9*, forward: 5′-atacccgggACTACAGACACGTGGACTTA-3′; reverse: 5′-attgcggccAACAAACAATTTATTACATG-3′. Simultaneously, the constructs of the PCR products and pCS107 vector were digested with EcoRI and XhoI, then purified and ligated with T4 DNA ligase (Thermo) at 22 °C. The ligation products were transformed into TG1 competent cells and herein the cells were spread on LB plates containing ampicillin (50 μg/mL). The *Hes5.9* fragment inserted into a vector was verified by colony PCR, the pCS107-*Hes5.9* plasmid was extracted from 2 mL overnight culture by SanPrep Kit (Sangon), and then using SP6 as a primer to sequence, the *Hes5.9* from pCS107-*Hes5.9* plasmid and the sequences were aligned with NCBI.

### 2.4. mRNA Synthesis and Microinjection

The plasmid pCS107-*Hes5.9* was linearized with ApaI (Takara), then capped mRNAs were synthesized using the mMESSAGE mMACHINE SP6 Kit (Ambion), and purified by MEGAclear Kit (Ambion). In brief, the following transcription reaction was carried out at room temperature: with 2 × NTP/CAP, 10× Reaction buffer, SP6 enzyme Mix, 1 μg linear template, and Nuclease-free water, the compound was incubated at 37 °C for 1 h, in sequent, the RNA was absorbed on the membrane in the filter cartridge, and then contaminants were washed away, lastly, mRNA was resuspended in a low salt buffer. The mRNA was bilaterally injected into the dorsal of the four-cell stage blastomere, meanwhile, the fluorescent dextran was co-injected as a lineage tracer.

### 2.5. Quantitative Reverse Transcription PCR (RT-qPCR)

Total RNA was extracted from *X. tropicalis* embryos according to the above-mentioned method, after synthesizing cDNA. RT-qPCR reactions were performed in triplicate for each sample, using a FastStart Universal SYBR Green Master (Roche) in CFX-Connect Real-Time System (BIO-RAD). The relative expression level of each target was normalized to the expression level of ornithine decarboxylase (*Odc*).

### 2.6. Whole-Mount in situ Hybridization

For the hybridization studies, the digoxigenin labeled antisense RNA probe of *Hes5.9* was prepared by linearizing the pCS107-*Hes5.9* plasmid with SmaI (Takara), and transcribing with T7 RNA polymerase (Promega). The different stages of embryos were collected and fixed in MEMFA (0.1 M MOPS, 2 mM EGTA, 1 mM MgSO_4_, 3.7% formaldehyde) for 2 h at room temperature, then these embryos were permeabilized by incubating them for about 15 min at room temperature in proteinase K (Roche; final concentration, 2.8 μg/mL), when the process of acetylation, fixation, and pre-hybridization was finished, the embryos were incubated in fresh hybridization buffer containing 0.5 ug/mL probe, and hybridized overnight at 60 °C. The embryos were washed with 2× saline sodium citrate (SSC), and 0.2× SSC at 60 °C, to remove the excess probe, then the embryos were washed twice with maleic acid buffer (MAB), MAB was replaced with blocking reagent (MAB, 2% Boehringer Mannheim blocking reagent, and 10% inactivated sheep serum), incubated for 2 h at room temperature; embryos were then incubated with antibody solution (Roche; anti-digoxigenin alkaline phosphatase (AP) antibody, 1:2000) overnight at 4 °C. The free antibody was removed by washing 3 × 30 min in MAB, before chromogenic reaction. We first washed the embryos 2 × 5 min at room temperature in alkaline phosphatase (AP) buffer, then incubated the embryos with BM purple (Roche), and when staining becomes apparent, embryos were fixed with MEMFA for 2 h at room temperature, then bleached with 30% hydrogen peroxide solution. Finally, the embryos were stored in 1× phosphate-buffered saline (PBS) for photographing.

### 2.7. Animal Cap and Dorsal Marginal Zone (DMZ) Elongation Assays

The embryos were injected with mRNA into the dorsal blastomeres, or MOs into ventral blastomeres at the four-cell stage embryos. Animal cap explants were excised at stage 8–9, and were cultured in 1× MBS with antibiotic, or together with 25 pg/mL recombinant human activin A (R&D) protein, until stage 17. DMZ explants were excised at stage 10.25 then cultured in 1× MBS with the antibiotic until stage 17.

### 2.8. RNA-Sequencing and Data Analyses

RNA sequencing was performed on the Illumina HiSeq2000 platform, and paired-end reads were mapped to the reference *Xenopus tropicalis* transcriptome annotation. HTSeq v0.6.1 was used to count the reads number mapped to each gene. Differential expression analysis of WT and *Hes5.9* overexpression was performed by using the DEGSeq R package (1.20.0). The P values were adjusted using the Benjamini and Hochberg method. Corrected *P*-value of 0.005 and log2 (fold change) of 1 were set as the threshold for significantly differential expression. Gene ontology (GO) enrichment analysis of differentially expressed genes was implemented by the GOseq R package. GO terms with corrected P-value less than 0.05 were considered significantly enriched by differentially expressed genes. KOBAS software was used to test the statistical enrichment of the differential expression genes in KEGG pathways (http://www.genome.jp/kegg/). The DEGs, GO, and KEGG were collected in Supplemental Excel files.

## 3. Results

### 3.1. Hes5.9 is Regulated by the Notch Signaling

In a systematic screen for differentially expressed transcripts under the influence of FGF signaling in *Xenopus tropicalis* embryos, we isolated a novel transcript during gastrulation. This transcript was denoted as *LOC733709*, and now suggested as *Hes5.9.* However, the biological function of this gene has not been characterized. According to the Ensembl database, *LOC733709* is located at the Scaffold GL172709.1, and is comprised of three exons and two introns, which encode a 155-amino acid protein (Supplementary Figure S1).

**Figure 1:**
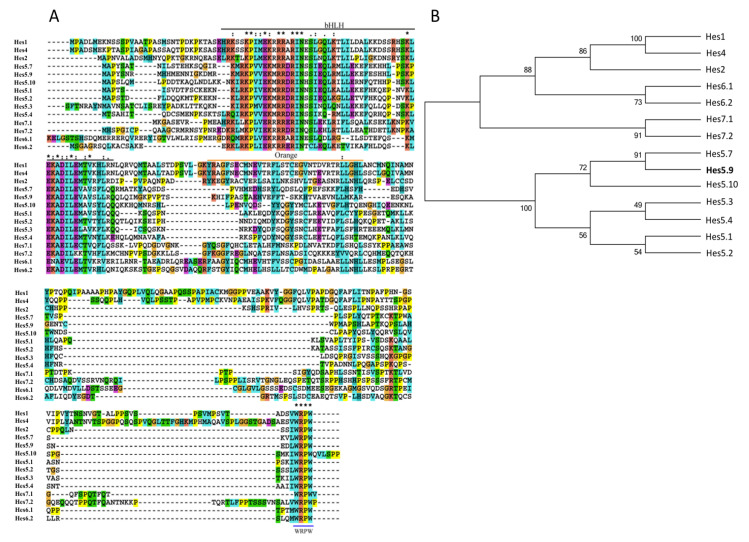
*Hes5.9* belongs to the Hes family. (**A**) Multiple sequence alignment of *Xenopus tropicalis Hes5.9* and related proteins. Comparison of the *Hes5.9* protein sequence with the related proteins that belong to the Hes family in *X. tropicalis*. There are three conserved domains: the bHLH domain, the orange domain, and the WRPW sequence at the carboxyl terminus between these proteins. The colors represent different similarity: violet is 100%, pink is 75% or more, and the blue is 50% or more. (**B**) Phylogenetic tree for *X. tropicalis Hes5.9* protein. The phylogenetic tree was constructed by using the comparison of full-length protein sequences.

**Figure 2:**
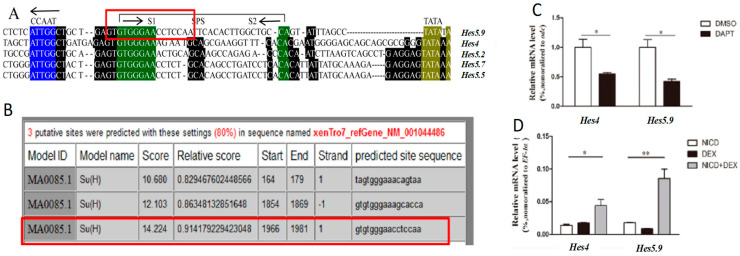
The *Hes5.9* is regulated by the Notch signaling. (**A**) Promoters of *Hes5.9*, *Hes4, Hes5.2, Hes5.7,* and *Hes5.5* show high and moderate homology of S1 and S2, respectively, in the SPS (green). All exhibit a conserved CCAAT motif (blue) and TATA box (yellow). (**B**) The predicted Notch binding sequences of *Hes5.9*. The expression patterns of *Hes4* and *Hes5.9* are affected by inhibition (**C**) or induction (**D**) of the Notch signaling, * *p* < 0.05; ** *p* < 0.01.

The Hes proteins consist of three conserved domains: the bHLH domain, the orange domain, and the C-terminal WRPW motif [29]. *Hes5.9* shows high identity to the *Hes* family members by multiple-sequence alignment (Figure 1A). The phylogenetic analysis further revealed that *Hes5.9* was closely related to *Hes5.7* (*Hes9.1*) and *Hes5* subfamily members (Figure 1B), which are the downstream genes of Notch signaling. Meanwhile, according to the gene locus, *Hes5.9* localizes in the *Hes5.3* cluster, consisting of *Hes5.3–Hes5.10*, therefore, *Hes5.9* was suggested to be possibly synchronously regulated by the Notch signaling. This was confirmed by transcriptional regulation sequence alignment (Figure 2A) and conserved Notch binding motif prediction (Figure 2B). *Hes5.9* contains paired Su(H) sites resembling an SPS, which are flanked by an inverse CCAAT motif, and proximal to the TATA box (Figure 2A). Meanwhile, a high scored Notch binding sequence located at −1966bp was further predicted by JASPAR (Figure 2B). When the embryos were treated with DAPT, an inhibitor of the Notch signaling, from stage 8, the mRNA expression level of *Hes5.9* was dramatically downregulated at stage 12 (later gastrula stage), resembling the Notch downstream gene *Hes4* (*Hairy2*) (Figure 2C). Conversely, embryos were microinjected with NICD-GR mRNA at the 4-cell stage, and the mRNA expression level of *Hes5.9* and *Hes4* were significantly upregulated at stage 12 with dexamethasone induction (Figure 2D). Collectively, *Hes5.9* is regulated by the Notch signaling, which might be a target gene of the Notch pathway.

### 3.2. Spatiotemporal Expression Pattern of Hes5.9 in Embryonic Development

**Figure 3:**
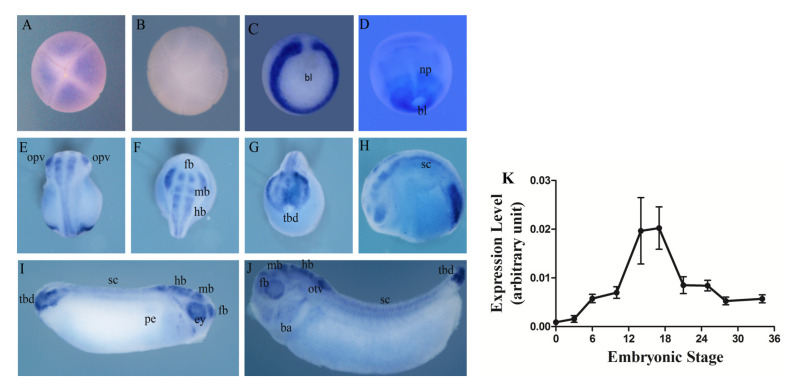
Spatiotemporal expression pattern of *Hes5.9* during embryonic development of *Xenopus tropicalis*. The spatial expression pattern of *Hes5.9* mRNA during development, which was examined by whole-mount in situ hybridization (WISH). (**A**) Cleavage stage 3, animal view; (**B**) Blastula stage 7, ventral view; (**C**) Early gastrula stage 10.5, vegetal view, dorsal to the top; (**D**) Early neurula stage 13, dorsal view, anterior to the top; (**E**–**G**) Mid neurula stage 19, dorsal view, anterior to the top in (**E**); anterior view, dorsal to the bottom in (**F**); posterior view, dorsal to the top in (**G**); (**H**) Late neurula stage 21, lateral view, dorsal to the top; (**I**) Early tailbud stage 28, lateral view, anterior to the right; (**J**) Late tailbud stage 35, lateral view, anterior to the left. (**K**) The temporal expression pattern of mRNA for *Hes5.9* during *X. tropicalis* embryonic development examined by RT-qPCR, and ornithine decarboxylase (*Odc*) was applied as a constant expression control. Abbreviations, bl: blastopore lip, np: neural plate, sc: spinal cord, ey: eye, tbd: tailbud, fb: forebrain, mb: midbrain, hb: hindbrain, opv: optic vesicle, otv: otic vesicle.

During *X. tropicalis* embryogenesis, the spatiotemporal expression pattern of *Hes5.9* was analyzed by WISH and RT-qPCR (Figure 3). It revealed that *Hes5.9* was gradually increased from egg to stage 10 (early gastrula stage), and climbed to a plateau during the neurula stages, while after stage 21 (late neurula stage), *Hes5.9* gradually declined (Figure 3K). Therefore, *Hes5.9* was suggested as a maternally expressed gene, which was confirmed by WISH in the 8-cell stage (Figure 3A). Meanwhile, *Hes5.9* was significantly upregulated from the gastrula stages to the neurula stages (Figure 3K) that indicated *Hes5.9* might be involved in gastrulation and tissue rudiment determination. The *Hes5.9* transcript was observed in the animal pole blastomeres at the early cleavage stage (Figure 3A), and lasted to the blastula stage (Figure 3K). However, no signal was detected in the vegetal pole (Figure 3B). At early gastrula stages, *Hes5.9* was expressed throughout the mesoderm except for the dorsal midline mesoderm (Figure 3C and Supplementary Figure S2). Then the signal narrowed toward the paraxial dorsal marginal zone (DMZ) at the early neurula stages and was expressed in the neural plate (Figure 3D). From the late neurula stage to the tailbud stage, the *Hes5.9* transcript was presented in the optic vesicle, otic vesicle, forebrain, midbrain, hindbrain, tailbud, and spinal cord (Figure 3E–J). Collectively, it showed that *Hes5.9* was mainly expressed in the mesoderm, neural primordium, and tailbud. Therefore, *Hes5.9* may play important roles in gastrulation, neural system development, and somitogenesis.

### 3.3. The FGF Signaling is Required for the Spatiotemporal Expression Pattern of Hes5.9 during Gastrulation

**Figure 4:**
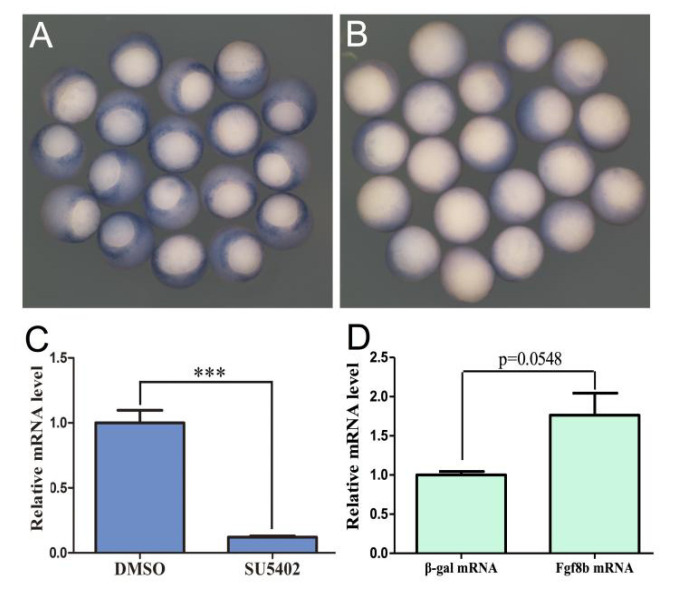
The mRNA expression level of *Hes5.9* is regulated by the FGF (Fibroblast growth factor) signaling. The FGFR (Fibroblast growth factor receptor) inhibitor SU5402 was utilized in embryos from stage 9 (pre-gastrula stage) to stage 11 (middle gastrula stage), while the control counterparts were treated with DMSO (Dimethyl Sulfoxide). The results of whole-mount in situ hybridization displayed: compared with DMSO-treated (**A**), the *Hes5.9* mRNA expression level was significantly declined by treatment with 20 μM SU5402 (**B**). A quantitative analysis of *Hes5.9* mRNA level was determined at stage 11 by RT-qPCR when the FGF signaling pathway was suppressed by SU5402 (**C**). (**D**) RT-qPCR analysis, the mRNA expression level of *Hes5.9* in stage 17 when each embryo was injected with 240 pg *Fgf8b* mRNA at the four-cell stage, and the *Hes5.9* expression was moderately increased. The values were normalized to *Odc*, *** *p* < 0.001.

**Figure 5:**
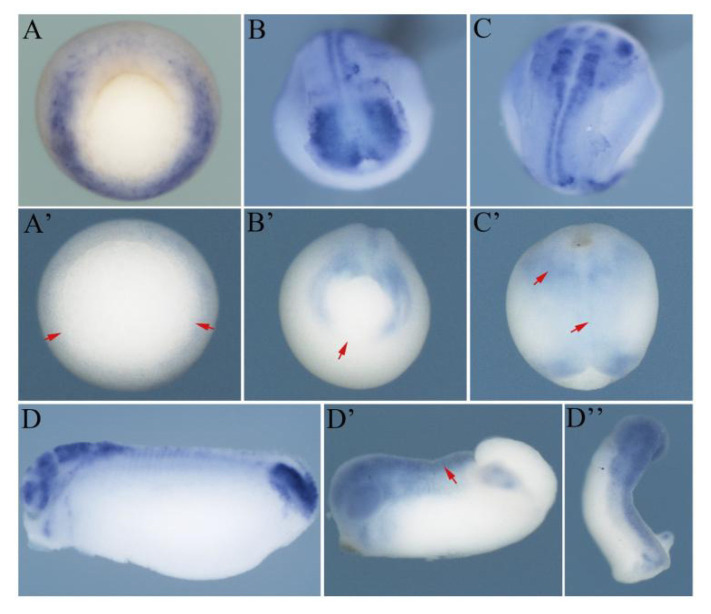
The spatiotemporal expression of *Hes5.9* was partially recovered after withdrawing SU5402. The mRNA expression pattern of *Hes5.9* was detected by WISH during the middle gastrula in mock embryos (**A**), and the embryos treated with SU5402 (**A’**), blastopore view with dorsal up; the late neurula mock embryos (**B**) and the embryos with withdrawn SU5402 (**B’**), posterior view with dorsal up; the late neurula mock embryos (**C**) and the embryos with withdrawn SU5402 (**C’**), dorsal view with head up; the tailbud stage, mock embryos (**D**) and embryos with withdrawn SU5402, lateral view (**D’**); and the embryos with withdrawn SU5402 with dorsal view (**D’’**). The red arrows indicate abnormal expression patterns of *Hes5.9*.

The *Hes5.9* was originally isolated from a systematic search for differentially expressed transcripts by inhibition of the FGF signaling during gastrulation in *X. tropicalis* embryos. Interestingly, the WISH results revealed that *Hes5.9* shared a similar expression pattern to *Myod*, a crucial downstream gene of the FGF signaling at the early gastrula stage [30,31]. Therefore, we speculated that *Hes5.9* was also regulated by the FGF signaling pathway. As shown in Figure 4, the *Hes5.9* was expressed throughout the mesoderm during gastrulation in the DMSO treated embryos (Figure 4A), but dramatically inhibited and dispersed around the blastopore by SU5402 treatment (Figure 4B). This was further validated by RT-qPCR (Figure 4C). In contrast, when upregulation of the FGF signaling by microinjecting embryos with *Fgf8b* mRNA at the 4-cell stage, the expression level of *Hes5.9* was moderately increased at stage 11 (Figure 4D). On other hand, we first inhibited FGF signaling by SU5402 and then recovered FGF signaling by withdrawing SU5402. As shown in Figure 5, after the withdrawal of SU5402, the transcription of *Hes5.9* was recovered at the neurula and the tailbud stages. However, the expression level was still much less than that in the mock embryos. Meanwhile, the spatial expression pattern of *Hes5.9* was severely interrupted at the neurula and the tailbud stages, which was divergently expressed in the primordium of the neural system, and vaguely among the somite and dorsum (Figure 5). Collectively, the mRNA expression levels of *Hes5.9* were downregulated by inhibition of the FGF signaling, and upregulated by overexpression of FGF8b or withdrawing the inhibition of the FGF signaling, which indicated that *Hes5.9* was also regulated by the FGF signaling pathway.

### 3.4. Knockdown of Hes5.9 Results in Defects on Gastrulation

**Figure 6:**
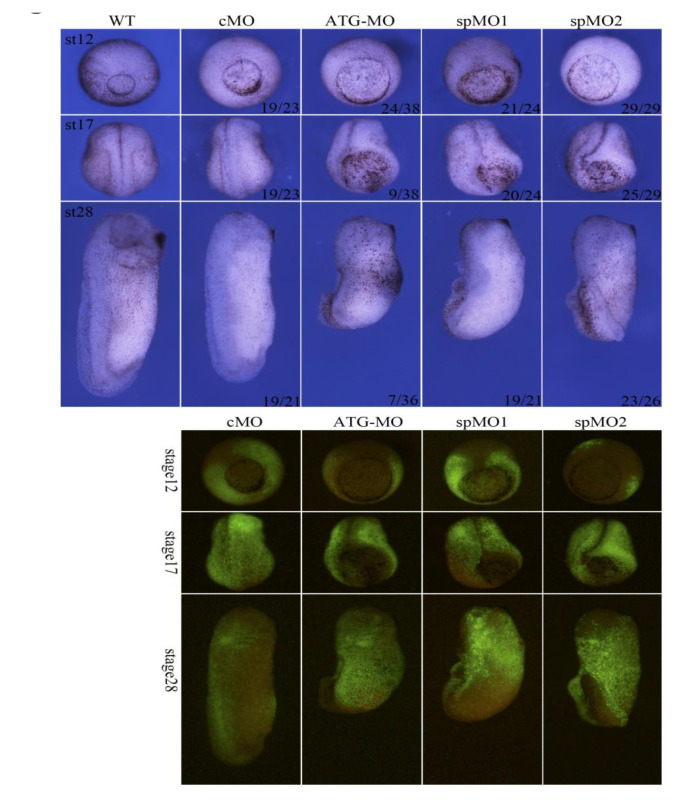
Knockdown of *Hes5.9* resulted in defective embryo morphogenesis. 10 ng MO/embryo was ventrally injected at the 4-cell stage. Meanwhile, fluorescent dextran was co-injected as a lineage tracer. cMO means control MO, ATG-MO targeting translational start site, while spMO1 and spMO2 binding to splicing sites (more details about scheme and efficiency are shown in supplementary data Figure S3).

To explore the functions of *Hes5.9* during early embryonic development in *X. tropicalis*, the embryos were dorsally microinjected at the 4-cell stage with 10 ng/embryo *Hes5.9* morpholino oligonucleotide (MO). However, they did not show obvious differences with the embryos injected with control MO. It was aforementioned that *Hes5.9* was highly expressed throughout the mesoderm, including the whole ventral mesoderm at the gastrula stage (Figure 3C), so we changed to knocked down ventral *Hes5.9*. It showed that three kinds of MOs performed efficiently (Figure 6), and the microinjection was successfully performed (Supplementary Figure S3), for the fluorescent tracers mostly localized among dorsal zone after the neurula and the tailbud stages (Figure 6). Intriguingly, the depletion of *Hes5.9* resembled the blocking of FGF signaling by SU5402. Although the blastopore lip formed normally, 81% (74/91) of embryos were delayed in blastopore closure by knocking down *Hes5.9* with MOs at late gastrula (Figure 6, st12). When neurulation initiated, 59% (54/91) of the *Hes5.9* knockdown embryos exhibited failures in blastopore and neural tube closure (Figure 6, st17). Meanwhile, the MOs at anti-splicing sites performed with better efficiency than at the anti-ATG translation start site. Although 92% of the embryos reached the tailbud stage, 89% (42/47) of the embryos were shortened in trunk and tail when injected with anti-splicing sites MOs (Figure 6). Therefore, depletion of ventral *Hes5.9* led to severe gastrulation defects, and further disturbing the development of the neural system, trunk, and tail.

### 3.5. Overexpression of Hes5.9 Causes Defects on Gastrulation

**Figure 7:**
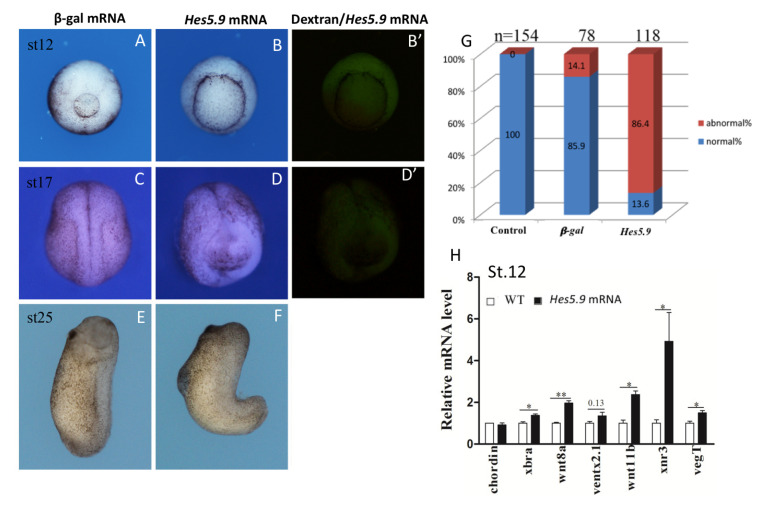
Overexpression of *Hes5.9* resulted in defective embryo morphogenesis. Embryos were bilaterally injected dorsally with 250 pg *Hes5.9* mRNA at the 4-cell stage, *β-gal* mRNA injected as a negative control, and dextran co-injected to indicate the inject site. (**A**–**F**) Ectopic expression of *Hes5.9* mRNA perturbed embryos normal development during gastrulation and neurulation, open blastopore and abnormal neural fold was observed. (**G**) Statistical analysis of abnormality. (**H**) The expression levels of the representative marker genes were determined by RT-qPCR at stage 12, * *p* < 0.05; ** *p* < 0.01.

The depletion of *Hes5.9* caused severe gastrulation defects (Figure 6), so what about the overexpression of *Hes5.9*. It showed that the blastopore lip formed normally, but 86.4% of embryos with *Hes5.9* ectopic expression exhibited gastrulation delaying and failed in blastopore and neural tube closure (Figure 7B,D,G). Most of the embryos survived to early tailbud stage (stage25) but exhibited a curved trunk or short tail (Figure 7F). This revealed that overexpression of dorsal *Hes5.9* perturbed gastrulation and neurulation, and mainly exhibited open blastopore and abnormal neural fold. As mesoderm cells play pivotal roles in morphogenetic movements, we then examined the expression patterns of the germ layer marker genes that were influenced by *Hes5.9* during gastrulation. As shown in Figure 7H, with overexpression of *Hes5.9,* pan-mesodermal marker *Xbra* and ventral mesodermal markers *Wnt8a, Xnr3, VegT,* and *Wnt11b* were remarkably upregulated, while *Chordin* and *Ventix2.1* were not significantly influenced (Figure 7H). Collectively, this indicated that *Hes5.9* might regulate cell fate decisions, thus modulating gastrulation and neurulation.

### 3.6. Hes5.9 Modulates the Elongation of Animal Cap and DMZ Explants

**Figure 8:**
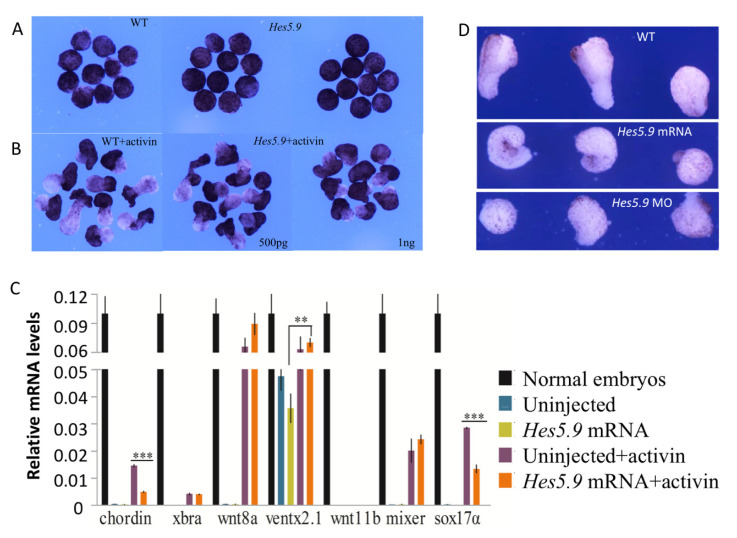
The *Hes5.9* inhibited cell fate specification and cell migration. A range of 500 pg-1 ng *Hes5.9* mRNA was injected into the destined dorsal cells at the 4-cell stage. Animal caps were dissected from wild type or *Hes5.9* overexpressed embryos at stage 8 and then cultured in 0.1× MMR without (**A**) or with (**B**) activin (25 pg/mL), and these explants were captured until the equivalent of stage 17. (**C**) The expression patterns of the marker genes were determined by RT-qPCR. (**D**) The dorsal marginal zone (DMZ) explants were dissected at stage 10.25 and a picture taken at stage 17, ** *p* < 0.01; *** *p* < 0.001.

Blastopore and neural tube closure are complex processes, while convergent extension and mesoderm migration are the fundamental morphogenetic movements that modulate these processes [32,33,34]. To address whether *Hes5.9* influences convergent extension and/or mesoderm migration, we performed the animal cap elongation assay from the embryos microinjected with *Hes5.9* or *β-gal* mRNA. The animal caps were dissected at stage 8 and then cultured to equivalent stage 17. Our data showed that the elongation of animal cap explants was dependent on the activin induction (Figure 8A,B). Meanwhile, the elongation rate is negatively related to the ectopic expression level of *Hes5.9* (Figure 8B). We also assessed the expression patterns of some representative marker genes in the animal caps. It showed that the expression of *chordin* and *Sox17a* were dramatically downregulated by overexpression of *Hes5.9* (Figure 8C). Furthermore, the elongation of DMZ explants was retarded dramatically by either knockdown or overexpression of *Hes5.9* (Figure 8D). Collectively, this revealed that *Hes5.9* modulated cell fate decision and mesoderm movement by regulating gene transcription and convergent extension.

### 3.7. Transcriptomics Analysis of Hes5.9 Ectopic Expression in Late Gastrula Stage

**Figure 9:**
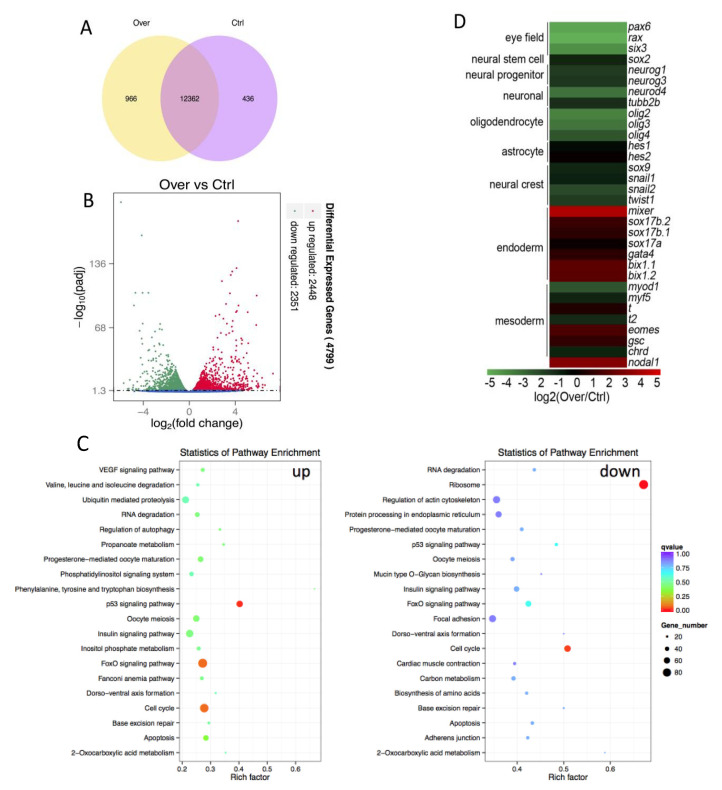
Transcriptomic analysis of *Hes5.9* overexpression at stage 12. (**A**) Total genes nominated in a Venn diagram. (**B**) Differential expressed genes by volcano diagram. (**C**) Differentially expressed genes were annotated in KEGG pathways. (**D**) Neurogenesis associated genes were displayed.

The embryos microinjected with *Hes5.9* or *β-gal* mRNA (as control) were harvested at stage 11.5. Thereafter, they were assessed by RNA-Seq and bioinformatics analysis. It showed that 12,362 transcripts were overlapped in the *Hes5.9-*overexpressed and control groups, while 966 and 436 transcripts were uniquely expressed in the *Hes5.9-*overexpressed and control groups, respectively (Figure 9A). Meanwhile, there were 4799 genes differentially expressed; whereas 2448 genes were upregulated, 2351 genes were downregulated with overexpression of *Hes5.9* (Figure 9B). The results further indicated that dorsal-ventral axis formation, cell cycle, apoptosis, p53 signaling pathway, and FoxO signaling pathways were influenced by ectopic expression of *Hes5.9* (Figure 9C and Supplementary Tables S1 and S2). Adherence junction, focal adhesion, and regulation of actin cytoskeleton were downregulated by ectopic expression of *Hes5.9*, which is consistent with the deficiency of animal cap and DMZ elongations (Figure 9C and Supplementary Tables S1 and S2). Intriguingly, we also found that the insulin signaling pathway and a battery of metabolism-associated genes were enriched by the ectopic expression of *Hes5.9* (Figure 9C and Figure S3). However, *Hes5.9* function in metabolism and development requires further elucidation.

Overexpression of *Hes5.9* significantly decreased the expression of neural progenitor markers *Neurog1* and *Neurog3*, neuronal markers *Neurod4* and *Tubb2b*, and eye field markers *Pax6*, *Rx*, and *Six3*, which indicates that *Hes5.9* could inhibit neural tissue formation. Meanwhile, overexpression of *Hes5.9* diminished the oligodendrocyte markers *Olig2, Olig3,* and *Olig4*, but not the astrocyte markers *Hes1* and *Hes2*, thus indicating that *Hes5.9* functions as a both neuronal and oligodendrocyte inhibitor. We also noticed that overexpression of *Hes5.9* diminished neural crest marker *Snail2* and *Twist1*. Besides, we found that overexpression of *Hes5.9* significantly upregulated endoderm markers such as *Mixer*, *Sox17b, Sox17a, Gata4,* and *Bix1*. We further checked mesoderm markers and found that the pan-mesoderm marker T *(Bra*), *Eomes,* and the dorsal mesoderm marker *Gsc* were also upregulated, while muscle mesoderm markers *Myod* and *Myf5* were downregulated by overexpression of *Hes5.9*. To explain this situation, we investigated the expression level of the meso-endoderm inducer *Nodal*. We found that *Nodal1* was significantly upregulated when *Hes5.9* was overexpressed in *Xenopus* embryos.

## 4. Discussion and Conclusions

In this study, we characterized the roles of *Hes5.9* in regulating cell fate decisions and cell migration in gastrulating *Xenopus tropicalis* embryos, which were regulated by the FGF and Notch signaling pathways during gastrulation. Results indicated the coordination of the FGF and Notch signaling pathways through fine-tuning of the expression pattern of *Hes5.9* during gastrulation.

We originally isolated a novel helix–loop–helix DNA binding domain protein (LOC733709), now suggested as *Hes5.9*, by screening the possible target genes of the FGF signaling during gastrulation. We characterized the roles of *Hes5.9* in embryogenesis, focusing on gastrulation in particular. Compared with humans, *Xenopus* has the same subfamilies (*Hes1–Hes7*), but more *Hes* genes, about 37. These *Hes* genes are involved in neurogenesis, somitogenesis *(Hes1, Hes5, Hes7, etc*.) [35], and midbrain-hindbrain boundary formation (*Hes7.1*) [36]. *Hes5* genes are located on the same chromosome, especially *Hes5.3–Hes5.10* located in the *Hes5.3* gene cluster. Most of these genes are downstream targets of the Notch signaling. As a *Hes5* gene member, *Hes5.9* contains conserved Notch signaling regulating promoter sequences. Moreover, the mRNA expression levels of *Hes5.9* were significantly influenced by either chemical or genetic interference of the Notch signaling (Figure 2). This evidence strongly indicates that *Hes5.9* would also be a target of the Notch signaling.

A previous study suggested that all 37 *Hes* genes, except *Hes2*, are zygotically expressed in early embryonic stages, and peak from the late gastrula to the late neurula stages (stage 12–20) [37]. Here, we found that *Hes5.9* was another maternally expressed *Hes* gene, which was highly expressed before the mid blastula transformation (MBT), and reached a peak during the neurula stages (Figure 3). Intriguingly, the expression patterns of *Hes5.9* are very different from *Hes5.7;* based on the Transcriptome Database (http://jason.chuang.ca/research/xenopus/refseq.html). Therefore, compared with other *Hes* genes, even *Hes5.7*, *Hes5.9* performs relatively unique roles during embryogenesis, especially during gastrulation.

The temporal expression pattern of *Hes5.9* mRNA revealed that *Hes5.9* was a maternally expressed gene, which was expressed throughout embryonic development and reached a plateau during the gastrula stage and the neurula stage. We also examined its spatial distribution by WISH, which suggested that *Hes5.9* asymmetrically localized along with the animal–vegetal axis, and was mainly detected at the animal pole. During gastrulation, *Hes5.9* is expressed predominantly throughout the mesoderm. As development proceeds, *Hes5.9* is detected in the neural tube, somites, tailbud, brain, neural crest, otic vesicle, and eyes (Figure 3 and Figure S2). The FGF signaling has been implicated during several phases of early embryogenesis [7], which contributes to the establishment of distinct types of mesoderm [38]. Meanwhile, the Notch signaling is involved early in the induction of the three germ layers [2], and later, playing important roles in somitogenesis and neural system development [39]. We further investigated the association of *Hes5.9* and the FGF signaling pathway during embryonic development by inhibiting FGFR via SU5402 [40] or overexpression of *Fgf8b* mRNA [41]. Here, we found evidence that the FGF signaling pathway is essential for the transcription of *Hes5.9* during gastrulation and neurulation (Figure 4 and Figure 5).

It has been reported that the FGF signaling pathway regulated both mesoderm migration and convergent extension movements [42,43,44]. The embryos treated with the SU5402 at the gastrula stage significantly decreased the expression level of *Hes5.9*, which was accompanied by delayed gastrulation and ultimately open blastopore (Figure 4 and Figure 5). Therefore, our results indicate that *Hes5.9* is regulated both by FGF and Notch signaling during gastrulation, and maybe an important gene for coordinating the FGF and Notch signaling during the gastrula and even the neurula stages. Although treatment with SU5402 caused abnormal embryonic development, we cannot exclude the possibility that the observed phenotype may be caused by the concomitant ectopic expression of *Hes5.9*. To investigate the character of *Hes5.9* during embryogenesis, we specifically knocked down *Hes5.9* by microinjecting with the morpholino antisense nucleotides that were selectively designed to block *Hes5.9* translation and splicing, respectively (Supplementary Figure S3). It showed that both gastrulation and neurulation were impaired. The embryos injected with *Hes5.9*-MO exhibit delayed mesoderm involution and failed to close the blastopore, which ultimately results in blastopore and neural tube open, and shorter axis embryos at later development (Figure 6). Similar phenotypes were obtained from ectopic expression of *Hes5.9* (Figure 7) that both mesoderm convergent extension and mesoderm migration were impaired by dorsally microinjecting *Hes5.9* mRNA (Figure 8). Meanwhile, we also found that downregulated genes were significantly enriched in adherence, and cytoskeletal remolding (Figure 9C and Figure S3). Consistently, *Hes* genes could also be required to control genes involved in cytoskeletal remodeling and the cell shape change, which are needed for initiating the migration itself [45,46]. That is also represented by the expression changes of genes, which are involved in the migration process, such as some extracellular matrix molecules and their receptors, cell adhesion molecules, and guide molecules. And the expression levels of those markers were also regulated by *Hes5.9*, either in whole embryos or explant tissues, further suggesting that *Hes5.9* plays important roles in cell fate specification. In general, these results suggest a crucial role of *Hes5.9* on gastrulation, that is *Hes5.9* may coordinate the FGF and Notch signaling to fine-tune the cell fate specification and morphogenetic movements [2].

Unexpectedly, we also found that the expression level of Xnr3, a target of the maternal Wnt/β-catenin pathway, was significant upregulated after overexpression of *Hes5.9* (Figure 7H). It seems contradictory to the previous report that Xnr3 was supposed to be inhibited by the Notch signaling [47]. However, Xnr3 is also reported to require the FGF signaling to induce cell elongation movements and thus allocating cells from the organizer [48]. Thus, it is possible that Xnr3 and *Hes5.9* coordinate gastrulation in different regions of the mesoderm, which, however, needs further investigation.

In conclusion, we characterized a novel *Hes* gene (*Hes5.9*) in *Xenopus tropicalis*. Our data suggest that *Hes5.9* plays important roles in gastrulation and neurulation, through regulating cell fate determination and convergent extension. Further exploration of the possible roles of *Hes5.9* in the coordination of the FGF and Notch pathways will bring new insights into the regulation of embryogenesis and organogenesis in future investigations.

## Supplementary Materials

The following are available online at https://www.mdpi.com/2073-4425/11/11/1363/s1, Figure S1: Genomic structure of the *Xenopus tropicalis Hes5.9* gene, Figure S2: The WISH of gastrulation embryos and transversal section, Figure S3: Three kinds of *Hes5.9*-MOs efficiently knockdown *Hes5.9*, Table S1: Ex.1 OvervsCtrl_all.DEG_KEGG_pa, Table S2: gene.description.
