# Supplementary material for: Hes5.9 Coordinate FGF and Notch Signaling to Modulate Gastrulation via Regulating Cell Fate Specification and Cell Migration in Xenopus tropicalis
Source: Genes (Basel). 2020 Nov 18;11(11):1363. doi: 10.3390/genes11111363 (PMC7699193; doi:10.3390/genes11111363)
Supplement: Supplementary file 1 [file genes-11-01363-s001.zip › Supplementary/Supplementary Figures.docx]

Supplementary Figure Legend


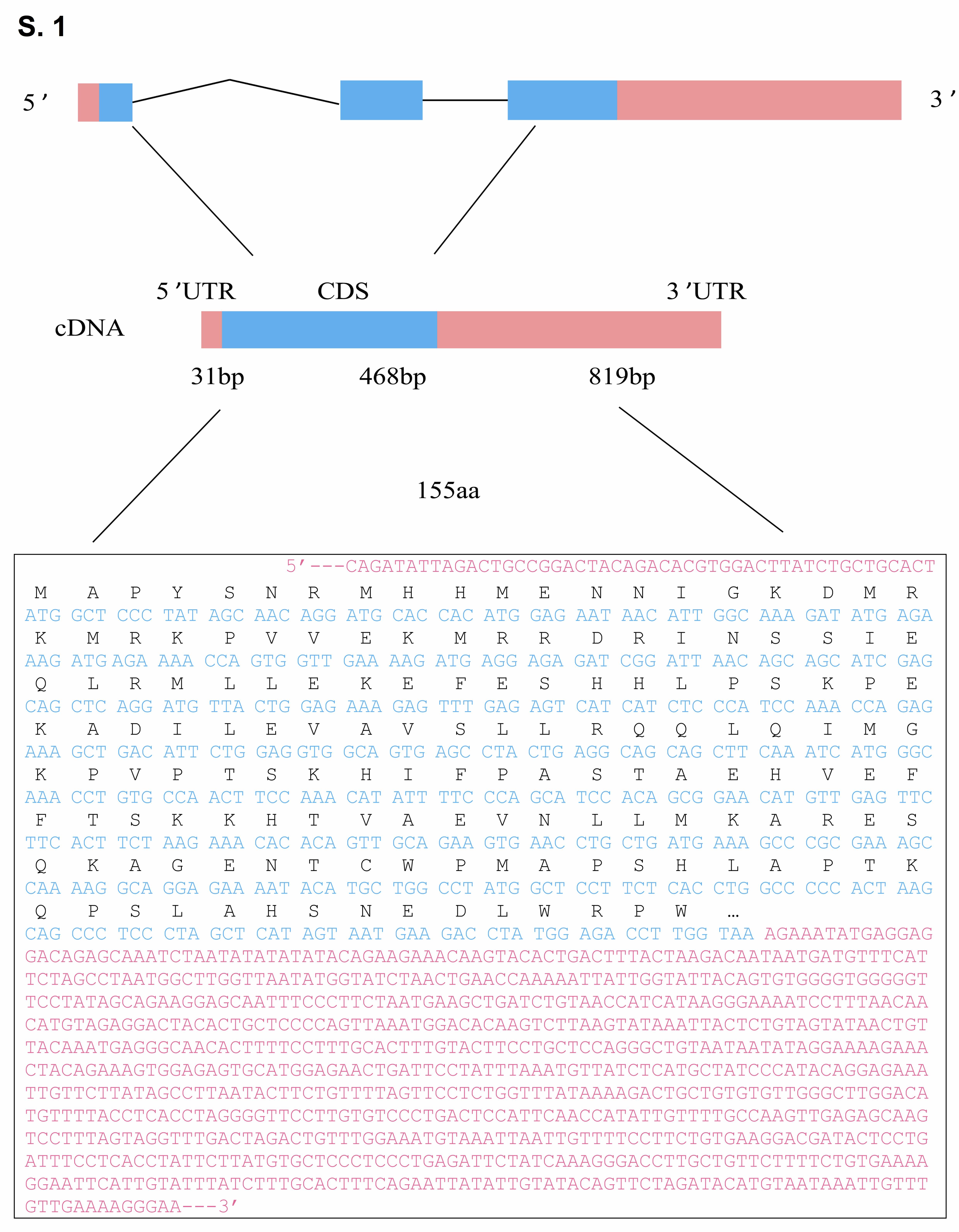


Figure S1. Genomic structure of the Xenopus tropicalis *Hes5.9* gene. The genomic structure of X. tropicalis *Hes5.9* is comprised of three exons and two introns. The full-length mRNA of *Hes5.9* consists with 5′ untranslated region, CDS, and 3′ untranslated region with a length of 31nt, 468nt and 819nt respectively. Nucleotide and amino acid sequences of *X. tropicalis* Hes5.9 are also shown in the box, which are composed of 1318 nucleotides and 155 amino acids respectively.


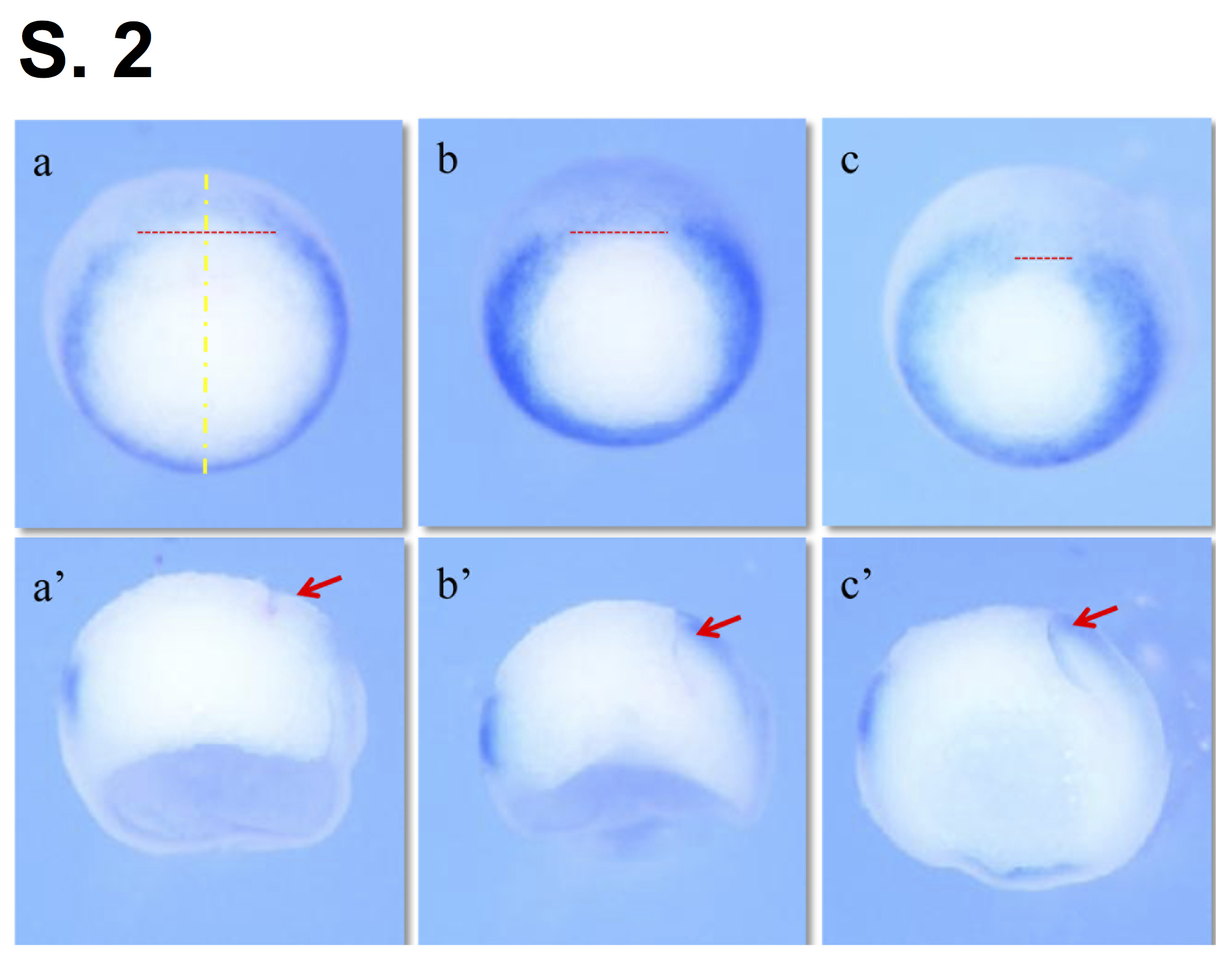


Figure S2. The WISH of gastrulation embryos and transversal section. **a**, **b**, **c** represent stage10.25，stage10.5 and stage11, while **a**’, **b**’, **c**’ were dissections of a, b, c, respectively. All the anatomized embryos were dissected along the midline (yellow).


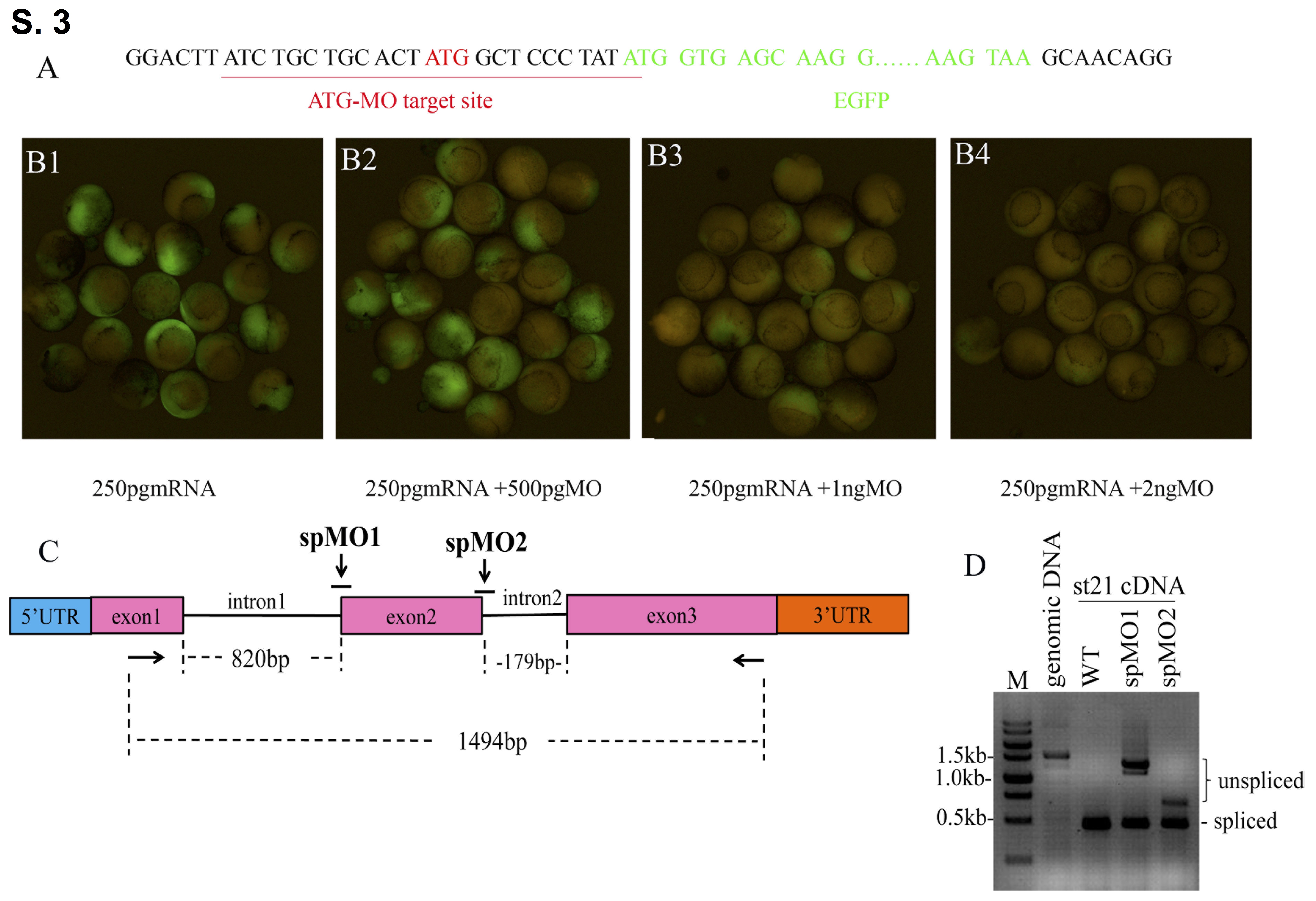


Figure S3. Three kinds of *Hes5.9*-MOs efficiently knockdown Hes5.9. (**A**) Constructing EGFP with parts of *Hes5.9* 5’ sequence, which is targeted by ATG-MO at translation starting site. (**B**) The expression patterns of GFP when co-injecting with different dosages of ATG-MO. (**C**) The schematic targets of the *Hes5.9* with MO1 and MO2. (**D**) The effectiveness of spMO1 and spMO2 that interrupting alternative splicing of *Hes5.9* was determined by PCR.
